# Association analysis of nine candidate gene polymorphisms in Indian patients with type 2 diabetic retinopathy

**DOI:** 10.1186/1471-2350-11-158

**Published:** 2010-11-10

**Authors:** Suganthalakshmi Balasubbu, Periasamy Sundaresan, Anand Rajendran, Kim Ramasamy, Gowthaman Govindarajan, Namperumalsamy Perumalsamy, J Fielding Hejtmancik

**Affiliations:** 1 Aravind Medical Research Foundation, Dr. G. Venkataswamy Eye Research Institute, Aravind Eye Hospital, #1, Anna Nagar, Madurai -625 020, India; 2 Ophthalmic Genetics and Visual Function Branch, National Eye Institute, NIH, Bethesda, MD, USA; 3 Retina Clinic, Aravind Eye Hospital, #1, Anna Nagar, Madurai -625 020, India; 4 Ophthalmic Genetics and Visual Function Branch, National Eye Institute, NIH, 5635 Fisher's Lane, Rockville, MD 20852, USA

## Abstract

**Background:**

Diabetic retinopathy (DR) is classically defined as a microvasculopathy that primarily affects the small blood vessels of the inner retina as a complication of diabetes mellitus (DM).It is a multifactorial disease with a strong genetic component. The aim of this study is to investigate the association of a set of nine candidate genes with the development of diabetic retinopathy in a South Indian cohort who have type 2 diabetes mellitus (T2DM).

**Methods:**

Seven candidate genes (*RAGE, PEDF, AKR1B1, EPO, HTRA1, ICAM *and *HFE*) were chosen based on reported association with DR in the literature. Two more, *CFH *and ARMS2, were chosen based on their roles in biological pathways previously implicated in DR. Fourteen single nucleotide polymorphisms (SNPs) and one dinucleotide repeat polymorphism, previously reported to show association with DR or other related diseases, were genotyped in 345 DR and 356 diabetic patients without retinopathy (DNR). The genes which showed positive association in this screening set were tested further in additional sets of 100 DR and 90 DNR additional patients from the Aravind Eye Hospital. Those which showed association in the secondary screen were subjected to a combined analysis with the 100 DR and 100 DNR subjects previously recruited and genotyped through the Sankara Nethralaya Hospital, India. Genotypes were evaluated using a combination of direct sequencing, TaqMan SNP genotyping, RFLP analysis, and SNaPshot PCR assays. Chi-square and Fisher exact tests were used to analyze the genotype and allele frequencies.

**Results:**

Among the nine loci (15 polymorphisms) screened, SNP rs2070600 (G82S) in the *RAGE *gene, showed significant association with DR (allelic P = 0.016, dominant model P = 0.012), compared to DNR. SNP rs2070600 further showed significant association with DR in the confirmation cohort (P = 0.035, dominant model P = 0.032). Combining the two cohorts gave an allelic P < 0.003 and dominant P = 0.0013). Combined analysis with the Sankara Nethralaya cohort gave an allelic P = 0.0003 and dominant P = 0.00011 with an OR = 0.49 (0.34 - 0.70) for the minor allele. In *HTRA1*, rs11200638 (G>A), showed marginal significance with DR (P = 0.055) while rs10490924 in LOC387715 gave a P = 0.07. No statistical significance was observed for SNPs in the other 7 genes studied.

**Conclusions:**

This study confirms significant association of one polymorphism only (rs2070600 in *RAGE*) with DR in an Indian population which had T2DM.

## Background

Diabetic retinopathy (DR) is the second leading cause of blindness due to retinal degeneration, contributing to an overall 4.8% blindness across the globe [1]. Clinically, diabetic retinopathy is characterized as basement membrane (BM) thickening, pericyte loss, endothelial cell (EC) dysfunction, microaneurysms, microvascular infarcts, and neovascularization in a patient with diabetic retinopathy [2,3]. Localized hypoxia has been implicated in this process, which can eventually lead to blindness. Although good glycemic control is considered to be a mainstay in preventing the vision loss, some patients with poor glycemic control do not develop retinopathy. This is well documented in the Diabetes Control and Complications Study (DCCT) and the United Kingdom Prospective Diabetes Study (UKPDS) [4,5].

Several bio-chemical pathways have been proposed to mediate the pathogenesis of DR. These include polyol accumulation, formation of advanced glycation end products (AGEs), oxidative stress, activation of protein kinase C, upregulation of matrix metalloproteinases, elaboration of growth factors and secretion of adhesion molecules [6,7]. Genetic as well as environmental factors may play a crucial role in understanding the differential susceptibility to DR. To date, markers at four chromosomal loci have been linked with DR. By multipoint sib-pair analysis, loci on chromosomes 3 and 9 showed suggestive evidence for linkage to DR in American Pima Indians with type 2 diabetes yielding lod scores of 1.36 and 1.46 respectively [8]. Two other recent studies also suggested loci on chromosomes 1 and 3, and 12 with suggestive support for a number of additional loci for DR in Mexican Americans [9,10].

DR shows a complex inheritance pattern, and genetic association studies are often used to identify the genetic factors influencing its development. In this context, polymorphic markers in or near several genes including aldose reductase (*AKR1B1*), the receptor for AGE (advanced glycation end products, *RAGE*), vascular endothelial growth factor (*VEGF*), intercellular adhesion molecule 1 (*ICAM-1*), pigment epithelium derived factor (*PEDF*), hemochromatosis (*HFE*), and alpha2beta1 integrin have been identified as being associated with DR [11-17]. However, with the exception of *VEGF*, *eNOS *(endothelial nitric oxide synthase), *IGF *(insulin like growth factor), *AKR1B1 *and *RAGE*, detailed investigations of most of these associations in the Indian population have not been carried out [13,18,19,21,23].

Therefore, the present study aims to evaluate SNPs in nine candidate genes previously reported to be associated with DR, including the receptor for advanced glycation end products (*RAGE*), pigment epithelium derived factor (*PEDF *or *SERPINF1*), aldose reductase (*AKR1B1*), erythropoietin (*EPO*), intercellular adhesion molecule 1 (*ICAM-1*), hemochromatosis (*HFE*), high temperature requirement A1 or serine peptidase (*HTRA1*), Complement Factor H (*CFH*) and age related maculopathy susceptibility 2 (*ARMS2*) to determine whether alleles of these markers are associated with DR in an Indian population.

## Methods

This study protocol was approved by both the Institutional Review board and Ethics Committee of Aravind Eye Hospital, Madurai, Tamil Nadu, India and Institutional Review board of the National Eye Institute (NEI), NIH, Bethesda, Maryland. Patients were recruited from the Aravind Eye Hospital and its clinics and the laboratory part of the study was carried out at the NEI in accordance with the tenets of Declaration of Helsinki.

### Enrollment of cases and controls

Patients who had T2DM (as defined by age of onset being later than 30 years of age) and exclusively used oral hypoglycemic agents were enrolled for this study from the patient population of the Aravind Eye Hospital and its outlying clinics. The criteria for diagnosis of diabetes were random blood sugars over 150 mgs% and HbA1C over 6%. A case-control design was used with two groups of diabetic patients: an unrelated ethnically matched group of patients with DR (n = 345) and a similar group of DNR (n = 359) were ascertained for the genetic association study. Patients with no signs of DR coupled with T2DM of 12-17 years duration were assigned to the DNR study group. Patients with proliferative diabetic retinopathy (PDR) as defined by eyes with definite neovascularization and/or vitreous/preretinal hemorrhages were included in the DR study group. Other patient details such as age, gender, body weight, duration of diabetes, family history of diabetes, other systemic illness, and treatment details were also documented (Table 1). They are essentially similar, although the mean duration of diabetes was slightly longer in the DNR individuals in enrollment group 2 (12 vs. 14 years). While the mean random blood sugars were slightly higher in the DR than DNR groups (212 vs. 188 and 222 vs. 102 respectively), the HbA1C values were similar (6.4 vs. 6.4 and 7.6 vs. 7.0 respectively). Statistically, the only significant differences in clinical characteristics between the DR and DNR groups overall were the percentage males (P = 0.05) and blood urea nitrogen (P = 0.002).

**Table 1:** Clinical Characteristics of the study subjects

	Patient enrollment 1		Patient enrollment 2		Kumaramanickavel et al.		
	DR (n = 345)	DNR (n = 359)	DR (n = 100)	DNR (n = 90)	DR (n = 100)	DNR (n = 100)	P (DR vs. DNR)*
Males (%)	70.0	58.0	56.7	57.6	79	64	0.05
Age (Years) (Mean± SD)	57 ± 9	59 ± 11	57 ± 8	58 ± 10	63 ± 7	62 ± 7	0.37
DM Duration ((Years) Mean ± SD)	14 ± 9	14 ± 9	12 ± 6	14 ± 5	17 ± 3	20 ± 5	0.42
Systolic Blood pressure (mmHg) (Mean± SD)	137 ± 18	135 ± 18	134 ± 17	128 ± 13	154 ± 26	143 ± 21	0.28
Diastolic Blood Pressure (mmHg) (Mean± SD)	83 ± 7	82 ± 7	83 ± 10	80 ± 10	86 ± 12	81 ± 9	0.28
Random Blood Sugar (mgs%)(Mean± SD)	212 ± 95	188 ± 85	222 ± 93	202 ± 80	N/A	N/A	0.80
HbA1C (%) (Mean± SD)	6.4 ± 1.6	6.4 ± 1.2	7.6 ± 1.7	7.0 ± 1.7	8.9 ± 0.74	8.2 ± 4.8	0.09
Serum creatinine (mgs%) (Mean± SD)	1.0 ± 0.7	0.8 ± 0.2	1.0 ± 0.4	0.8 ± 0.3	N/A	N/A	0.32
Blood urea (mgs%) (Mean± SD)	32 ± 14	25 ± 8	32 ± 11	27 ± 8	N/A	N/A	0.002

N/A not available

*trend test carried out on combined enrollment 1 and enrollment 2 data sets.

All patients were subjected to a clinical evaluation comprising a fundus examination by a binocular indirect ophthalmoscope and a slit lamp biomicroscopic examination with a 90 D lens in the retina clinic of Aravind Eye Hospital, Madurai, India. In addition, all patients underwent 5-field 50° color fundus photography.

The second (confirmatory) group of patients and control individuals including DR (n = 100) and DNR (n = 90) were similarly recruited through the Aravind Eye Hospital and its clinics and met the same diagnostic criteria as the first (exploratory) group. Ascertainment and diagnosis of patients seen at the Medical Research Foundation, Sankara Nethralaya in Chennai, India were described previously in detail [21]. Briefly, consecutive unrelated type II diabetic patients seen at the Sankara Nethralaya were tested for blood glucose and glycosylated Hb levels and a medical history including their sex, weight, duration and treatment of diabetes. Enrollment of cases and controls at the Sankara Nethralaya Hospital has been previously described and essentially met the same criteria as those used at the Aravind Eye Hospital [21].

### Candidate gene and single nucleotide polymorphism selection

A total of 14 SNPs and one dinucleotide repeat polymorphism in nine candidate gene regions were selected for the present study. These genes were all reported to be associated with DR in at least one population or are logical candidate genes based on the current understanding of the pathogenesis of DR. The selected SNPs are in the promoter region, 5'UTR region, or coding regions of candidate genes. The primers for selected SNPs and their corresponding genes are listed (Table S1, Additional File 1). The primers were designed for each SNP using Primer III, http://frodo.wi.mit.edu/, except for a subset of SNPs (-374 T/A,-429 T/C and rs2070600 in *RAGE*, rs35839483 in *ALR*, rs5498 in *ICAM-1*, rs1136287, rs58697961, rs12150053 and rs12948385 in *PEDF *and rs1617640 in *EPO*) for which the primers were chosen from previous studies [12,14,15,23,41].

### Genotyping

Total genomic DNA was extracted from the peripheral blood leukocytes by a salt precipitation method after obtaining written informed consent from all the participants [22]. The SNPs in the *RAGE *(-374 T/A, -429 T/C and rs2070600), *PEDF *(rs12150053, rs12948385, rs58697961 and rs1136287), *AKR1B1 *(rs35839483), *EPO *(rs1617640), *HTRA1 *(rs11200638), *ICAM *(rs5498), *HFE *(rs1800562), *CFH *(rs1061170 and rs3753394) and *ARMS2/LOC387715 *(rs10490924) were chosen on the basis of their association with DR in previous studies. SNPs were amplified by the polymerase chain reaction (PCR), (Thermocycler 9700; Applied Biosystems, Inc. [ABI], Foster City, CA). PCR reactions were performed in 20-μL reaction volumes containing 10 mM Tris HCl (pH 8.9), 50 mM KCl, 1.5 mM MgCl_2_, 0.2 μM of each primer, 250 μM of each dNTP, 50 ng of genomic DNA, and 0.5 units of *Taq *thermostable DNA polymerase (ABI, Foster city, CA, USA). Cycling parameters were 5 minutes at 95°C, followed by 35 cycles of 30 seconds at 95°C, 30 seconds at the annealing temperature (T_m_) of the primers (52°C-63°C, see Table S1, Additional File 1), and 45 seconds to 1 minute at 72°C, with a final 10-minute extension at 72°C.

Genotyping of rs1136287, rs58697961, rs12150053 and rs12948385 in *PEDF*, rs11200638 in *HTRA1*, promoter polymorphisms in *RAGE *(-429T/C, -374 T/A) were performed by direct sequencing (BigDye terminator chemistry, ver. 3.1; ABI) using an automated DNA sequencer (Prism 3130xl; ABI, Foster city, CA, USA). Genotyping of rs1617640 in *EPO *was carried out by Taqman real time PCR with an ABI 7900HT Fast Real-Time PCR System (ABI, Foster city, CA, USA). Genotyping of the *ICAM-1 *SNP rs5498 (K469E in exon 6), the *HFE *SNP rs1800562 (C282Y in exon 3), the *CFH *SNPs (rs1061170 and rs3753394), the *ARMS2/LOC387715 *gene SNP rs10490924 and rs2070600 in *RAGE *were carried out by SNaPshot PCR (ABI3100 Genetic Analyzer, ABI, Foster city, CA, USA) except for the cases studied by Kumaramanickavel et al. [21] which were genotyped by *AluI *RFLP analysis. Genotyping of rs35839483 in *AKR1B1 *was performed using the primers described by Kumaramanickavel et al. [23] with an M13 fluorescently-labeled tail primer followed by electrophoresis on an ABI3130xl genetic analyzer (ABI, Foster city, CA, USA). It should be noted that this technique offsets the allele sizes by 19 bp. All genotypes were interpreted by 2 readers blinded to the sample phenotypes, and the results were correlated and repeated if disagreements could not be unambiguously resolved. All markers are in HWE overall and for cases and controls with the exception of rs2070600, which shows slight deviation ( p > 0.001) only in the DR samples in the confirmatory data. The confirmatory data set overall and DNR set as well as all groups in the exploratory and combined ARAVIND data sets are in HWE for all markers tested.

### Statistical Analysis

Chi-square, and Fisher exact tests were used to test the allelic and genotypic associations of all the SNPs, and Hardy-Weinberg equilibrium of each SNP in control as well as in affected individuals was also examined using a Χ^2 ^test, all as implemented in the Exemplar program (Sapio Sciences, Baltimore, MD) or the Golden Helix SVS software suite 7 (Golden Helix, Bozeman, MT). Odds ratio, relative risk, and call rate were also calculated using the same programs. Because this is a directed search of candidate genes with a presumed high *a priori *probability of being associated a P-value < 0.05 was considered to be statistically significant. Corrections for multiple testing were done by the Bonferroni method where indicated. Association of clinical variables with DR vs. DNR was carried out using the trend test and calculated for the combined exploratory and confirmatory data sets.

## Results

The clinical characteristics of the study subjects are shown in Table 1. In this study, patients with PDR as defined by eyes with definite neovascularization and/or vitreous/preretinal hemorrhages and more severe hemorrhages were selected for the DR study group and patients with no signs of DR coupled with type 2 diabetes of at least 12 years duration were selected for diabetic without retinopathy (DNR) study group. The criteria for diagnosis of diabetes were random blood sugars over 150 mgs% and HbA1C over 6%. The clinical characteristics such as random blood sugars, HbA1C, blood pressure, and duration of diabetes were essentially similar in both groups, although the DR and DNR exploratory groups both had somewhat longer duration of diabetes in the report by Kumaramanickavel et al. [21] than the confirmatory groups (17 and 20 vs. 14 and 14 years, respectively). Of the clinical characteristic recorded only percentage males (P = 0.05) and blood urea nitrogen (P = 0.002) were significantly associated with DR. None of the clinical characteristics other than DR were associated with any tested genotypes.

Among the 15 SNPs studied, only one, rs2070600 (G>A) in exon 3 of *RAGE*, which leads to a change of amino acid glycine to serine at codon 82 displays significant association with DR in the initial screening group (Table 2). The G allele (P = 0.016), the homozygous GG (P = 0.012), and the heterozygous GA (P-0.011) genotypes are significantly associated with DR. Individuals with the homozygous GG genotype of this SNP have 1.78 times higher risk of developing DR (OR = 1.78: 95% CI-1.13 - 2.82). A dominant model also shows association with DR (P = 0.012, Table 3), with the A allele in either heterozygous of homozygous state giving an odds ratio (OR) = 0.56 with 95% CI = 0.35 - 0.89. In contrast, the other *RAGE *gene polymorphisms tested, -374 T/A and -429 T/C, do not show any significant association in this population. No other SNPs previously reported to show association could be confirmed in this population, with the possible exceptions of rs11200638 (G>A) in *HTRA1 *and the LOC387715 SNP rs10490924, which show marginally significant associations with DR (allelic P = 0.055 and P = 0.07, respectively).

**Table 2:** Allele and Genotype distribution of Nine candidate gene polymorphisms in the study

Gene	SNP	Allele	DR	DNR	Odds ratio (95% CI)	P	Genotype	DR (n = 345)	DNR (n = 359)	Odds ratio (95% CI)^	P
RAGE	(-374 T/A)	T	98.10%	98.52%	0.77 (0.27-2.15)	0.62	TT	96.21%	97.05%	0.76 (0.27-2.13)	0.62
		A	1.89%	1.48%			TA	3.79%	2.95%	1.29 (0.46-3.63)	0.62
	(- 429T/C)	T	86.26%	83.76%	1.22 (0.84-1.76)	0.29	TT	74.88%	71.31%	1.19 (0.78-1.82)	0.39
		C	13.74%	16.24%			TC	22.75%	24.89%	0.88 (0.57-1.37)	0.59
							CC	2.37%	3.80%	0.61 (0.20-1.86)	0.38
	rs2070600	G	95.1%	91.9%	1.7 (1.1-2.6)	0.016*	GG	90.4%	84.1%	1.78 (1.13- 2.82)	0.012*
		A	4.9%	8.1%			GA	9.3%	15.6%	0.55 (0.35-0.88)	0.011*
							AA	0.3%	0.3%	1.04 (0.06 -16.7)	0.97*
PEDF	rs12150053	T	77.86%	76.06%	1.10 (0.80-1.51)	0.52	TT	60.95%	58.12%	1.12 (0.76 -1.64)	0.54
		C	22.14%	23.94%			TC	33.81%	35.90%	0.91 (0.61-1.34)	0.64
							CC	5.24%	5.98%	1.04 (0.06-1.95)	0.73
	rs12948385	G	78.33%	80.17%	0.89 (0.64-1.23)	0.49	GG	62.38%	66.67%	0.82 (0.56-1.22)	0.34
		A	21.67%	19.83%			GA	31.90%	27%	1.26 (0.84-1.90)	0.25
							AA	5.71%	6.33%	0.89 (0.40-1.96)	0.78
	rs58697961	G	50.47%	51.90%	0.94 (0.72-1.22)	0.67	GG	27.01%	26.58%	1.02 (0.67-1.55)	0.91
		A	49.53%	48.10%			GA	46.92%	50.63%	0.86 (0.59-1.24)	0.43
							AA	26.07%	22.78%	1.19 (0.77-1.84)	0.41
	rs1136287	C	58.17%	54.03%	1.18 (0.90-1.54)	0.21	CC	34.13%	28.40%	1.30 (0.87-1.95)	0.19
		T	41.83%	45.97%			CT	48.08%	51.27%	0.88 (0.60-1.27)	0.5
							TT	17.79%	20.33%	0.84 (0.52-1.36)	0.49
HTRA1	rs11200638	G	61.7%	70%	0.68 (0.45-1.01)	0.055	GG	39.34%	47.68%	0.64 (0.37-1.1)	0.11
		A	38.3%	30.0%			GA	46.92%	43.04%	1.18 (0.69-2.04)	0.53
							AA	13.74%	9.28%	1.86 (0.8-4.3)	0.14
EPO	rs1617640	G	67.30%	67.72%	0.98 (0.74 -1.29)	0.89	GG	43.60%	43.88%	0.98 (0.68-1.43)	0.95
		T	32.70%	32.28%			GT	47.39%	47.68%	0.98 (0.68-1.43)	0.95
							TT	9%	8.44%	1.07 (0.55-2.07)	0.83
ICAM	rs5498	A	53.42%	51.91%	1.06 (0.81-1.39)	0.66	AA	30%	27.66%	1.12 (0.73-1.70)	0.59
		G	46.58%	48.08%			AG	46.84%	48.51%	0.93 (0.63-1.37)	0.73
							GG	23.16%	23.83%	0.96 (0.61-1.51)	0.87
CFH	rs3753394	T	72.03%	69.49%	1.13 (0.83-1.53)	0.42	TT	55.36%	49.15%	1.28 (0.86-1.89)	0.21
		C	27.97%	30.51%			CT	33.33%	40.68%	0.72 (0.48-1.09)	0.12
							CC	11.30%	10.17%	1.12 (0.60-2.10)	0.71
	rs1061170	C	69.86%	72.25%	0.89 (0.66 - 1.19)	0.43	CC	48.33%	50%	0.93 (0.64-1.35)	0.72
		T	30.14%	27.75%			CT	43.06%	44.49%	0.94 (0.64-1.37)	0.76
							TT	8.61%	5.50%	1.61 (0.77-3.38)	0.19
LOC387715	rs10490924	T	62.57%	68.43%	0.77 (0.57 - 1.02)	0.07	TT	38.55%	47.03%	0.70 (0.47 - 1.04)	0.08
		G	37.43%	31.57%			TG	48.04%	42.80%	1.23 (0.83 - 1.82)	0.28
							GG	13.41%	10.17%	1.36 (0.74 - 2.49)	0.3

Chi-square test was used to compare the genotype and allele frequencies between cases and controls.

The SNPs are in Hardy-weinberg equilibrium.

*P-Value < 0.05 is considered to be statistically significant.

^The odds ratio for one genotype was calculated against the other two genotypes combined.

**Table 3:** Analysis of RAGE under various models in the exploratory and confirmatory sets

Gene	SNP	Allele	DR	DNR	Odds ratio (95% CI)	P	Model	Odds ratio (95% CI)	P
RAGE	rs2070600	G	95.1%	91.9%	1.7 (1.1 -2.6)	0.016^2^	SNP		0.04^2^
		A	4.9%	8.1%	0.59 (0.38-0.91)		Dominant	OR = 0.56 (0.35 - 0.89)^1^	0.012^2^
							Additive	R = -0.091	0.016^3^

^1^(AA,AG)/AA ^2^χ^2^, P-Value < 0.05 is considered to be statistically significant. ^3^Trend, P-Value < 0.05 is considered to be statistically significant.									
									
**Genotype and allele frequencies of RAGE in the confirmatory set:**									

**Gene**	**SNP**	**Allele**	**DR**	**DNR**	**Odds ratio** **(95% CI)**	**P**	**Model**	**Odds ratio/R** **(95% CI)**	**P**

RAGE	rs2070600	G	96.5%	92.2%	1.78 (1.22 -2.60)	0.068	SNP		0.035^2^
		A	3.5%	7.8%	0.54 (0.38-0.76)		Dominant	OR = 0.34 (0.13 - 0.94)^1^	0.032^2^
							Additive A	R = -0.129	0.07^3^

*includes the exploratory, confirmatory sets and that of Kumaramanickavel et al.

^1^(AA,AG)/AA

^2^χ^2^, P-Value < 0.05 is considered to be statistically significant.

^3^Trend, P-Value < 0.05 is considered to be statistically significant (not corrected for multiple testing).

When association of rs2070600 is tested in the confirmatory group the association is confirmed. Although the allelic association test gives only a suggestive P = 0.068 (A allele OR = 0.54: 95% CI = 0.38 - 0.76), the whole SNP P = 0.035 and a dominant model for the A allele gives a P = 0.032 with an OR = 0.34 (95% CI = 0.13 - 0.94). Combining the two groups for analysis continues to show significant association of rs2070600 with DR (allelic P < 0.003). Similarly, the genotypic test (P = 0.003), the dominant model (P = 0.0013, OR = 0.51, 95% CI = 0.34 - 0.82) and the additive model (P = 0.003) all show significant association (Table 4).

**Table 4:** Analysis of combined sets under various models

Genotype and allele frequencies of RAGE gene polymorphism rs2070600 in the exploratory and confirmatory sets combined:									
**Gene**	**SNP**	**Allele**	**DR**	**DNR**	**Odds ratio** **(95% CI)**	**P**	**Model**	**Odds ratio** **(95% CI)**	**P**

RAGE	rs2070600	G	95.4%	92%	1.8 (1.2 -2.7)	0.003^2^	SNP		0.003^2^
		A	4.6%	8%	0.55 (0.37-0.82)		Dominant	0.51 (0.34 - 0.77)^1^	0.0013^2^
							Additive	R = -0.0997	0.003^3^
									
Genotype and allele frequencies of RAGE in all sets combined*:									

**Gene**	**SNP**	**Allele**	**DR**	**DNR**	**Odds ratio** **(95% CI)**	**P**	**Model**	**Odds ratio/R** **(95% CI)**	**P**

RAGE	rs2070600	G	95.2%	91.4%	1.78 (1.22 -2.60)	0.0003*	SNP		0.0003^2^
		A	4.8%	8.6%	0.54 (0.38-0.76)		Dominant	0.49 (0.34 - 0.70)^1^	0.00011^2^
							Additive A	R = -0.10548	0.00047^3^

*includes the exploratory, confirmatory sets and that of Kumaramanickavel et al.

^1^(AA,AG)/AA

^2^χ^2^, P-Value < 0.05 is considered to be statistically significant.

^3^Trend, P-Value < 0.05 is considered to be statistically significant (not corrected for multiple testing).

The *RAGE *gene was selected as a candidate based on previous analysis showing association in an Indian population by Kumaramanickavel et al [21]. Because of the ethnic and clinical similarities between the ARAVIND and Sankara Nethralaya study populations (Table 1) and because they represented the only two studies examining *RAGE *gene polymorphisms in the Indian population, we decided to combine data from the two studies in a single analysis. It was noted that our results for the *RAGE *gene were remarkably similar to those reported by Kumaramanickavel et al. [21] with major allele frequencies of .95 vs. .95 and .92 vs. .89 for the DR and DNR groups, respectively. The combined results once more confirmed association of the G allele with DR: allelic P < 0.0003 with the A allele OR = 0.54 (0.38 - 0.76). The whole SNP association is also significant with a P = 0.0003, as are the results of analysis under specific models with the A allele dominant P = 0.00011 with an OR = 0.49 (95% CI = 0.34 - 0.7) and the additive model P = 0.00047 (Table 4).

None of the remaining SNPs, including, four SNPs rs1136287 (C/T), rs58697961 (G/A), rs12150053 (T/C) and rs12948385 (G/A) in the *PEDF *gene, rs1617640 (G/T) in *EPO*, rs1800562 (G/A) in *HFE*, rs5498 (A/G) in *ICAM*, rs1061170 and rs3753394 in *CFH*, rs10490924 in *ARMS2/LOC387715 *and rs35839483 (CA)n in *AKR1B1 *showed significant association with DR. The SNP rs1800562 (G/A) in *HFE *was found not to be polymorphic in the study groups. Additionally, 12 alleles 138 bps-162 bps in rs35839483 (CA)n were identified. The allele frequencies of the *AKR1B1 *(CA)n dinucleotide repeat polymorphism are given in Table 5. None of the alleles show significant association with DR in this population.

**Table 5:** Allele frequencies of ALR2 (CA)n polymorphism

Allele	DR (n = 422)	DNR (n = 474)	Odds Ratio (95% CI)	P-Value
138	0	3 (0.63%)	∞	0.1<p<0.25
140	4 (0.94%)	8(1.68%)	0.55 (0.16 -1.86)	0.5<p<0.25
142	1 (0.23%)	0	∞	0.5<p<0.25
146	3 (0.71%)	0	∞	0.1<p<0.05
148	20 (4.73%)	16 (3.37%)	1.424 (0.72 -2.78)	0.5<p<0.25
150	73 (17.29%)	65 (13.71%)	1.316 (0.91 -1.89)	0.75<p<0.5
152	189 (44.78%)	216 (45.56%)	0.96 (0.74 -1.26)	0.9<p<0.75
154	55 (13.03%)	65 (13.71%)	0.94 (0.64 -1.38)	0.9<p<0.75
156	41 (9.71%)	43 (9.07%)	1.07 (0.68 -1.69)	0.9<p<0.75
158	23 (5.45%)	39 (8.22%)	0.64 (0.37 -1.09)	0.1<p<0.25
160	11 (2.60%)	19 (4%)	0.64 (0.30 -1.36)	0.1<p<0.25
162	2 (0.47%)	0	∞	0.1<p<0.25

## Discussion

The present study evaluates the potential association of fifteen polymorphisms in nine previously identified DR candidate genes with type 2 diabetes patients in a South Indian population. In this case-controlled study only one SNP, rs2070600 in the *RAGE *gene, could be shown to be significantly associated with retinopathy in the presence of DR. Because of the relatively small number of patients and controls analyzed, this study cannot necessarily exclude the possibility of alleles of the remaining SNPs showing some association with DR, especially those in HTRA1 and LOC387715. However, it is unlikely that the level of association would be greater than the 95% confidence limits of the odds ratios shown in Table 3, which are generally less than 1.5 for allelic association with the exception of the *RAGE *related SNPs, and less than 2-2.5 for the genotype odds ratios. Finally, it is possible that associations seen in other populations might not be present in southern Indians because of different population histories, which could alter haplotype block structure and thus non-causative allelic association, and possibly differing genetic and environmental contributions to the disease risk in various ethnic and geographic groups. In addition, this study did not address the use of oral hypoglycemic agents on the development of DR directly. However, since the main effects of these agents would be expected to result from their enhancement of glycemic control, to some extent one might expect their effects to be reflected in the fasting blood glucose and HbA1C levels, which were similar in the DR and DNR subjects.

*RAGE *gene polymorphisms are attractive candidates to influence DR because of pathophysiological data correlating retinopathy and advanced glycation end products (AGEs). *RAGE*, which maps to chromosome 6p21.3, is a member of the immunoglobulin super family. AGEs result from the non-enzymatic glycation of proteins and lipids. They are found at increased levels in diabetes and can lead to increased oxidative stress and receptor-mediated activation and secretion of various cytokines [24,25]. Accumulation of AGEs has been suggested to contribute to vasculopathy by increasing retinal endothelial cell permeability [26]. Specific AGE-binding receptors include the AGE-receptor complex, the macrophage scavenger receptors, and the receptor for AGE (*RAGE*). A Gly82Ser polymorphism in *RAGE *is potentially interesting since it occurs at a predicted N-linked glycosylation motif in the AGE binding site, thereby influencing AGE-RAGE interactions [12]. Kumaramanickavel et al. [21] previously reported that this polymorphism is associated with decreased risk for the development of DR in a Southern Indian population essentially similar to the one studied here. However, additional studies investigating the same locus did not show significant association for this polymorphism in Chinese and Japanese populations [20,27], and a recent study showed association of the A allele (serine) with DR in a Han Chinese population [28]. Association of the opposite allele of a polymorphism with disease has been reported before, and an additional example with an elegant theoretical analysis of possible causes for this has been presented by Lin et al. [29,30]. As also suggested by Zhang et al. [28] in their report, this might represent either different molecular pathogenesis of the disease in the two populations or simply a different population history resulting in different haplotype structure of the associated region. A particularly interesting example is that of the *LOXL1 *polymorphisms rs3825942 and rs1048661, which are in linkage disequilibrium and both show association with exfoliative glaucoma (XFG) [31,32]. While the G allele of rs3825942 is associated with XFG in both Caucasian and Japanese populations, the G allele of rs1048661 (GG haplotype of rs1048661 and rs3825942) is associated with XFG in Caucasians but the T allele (TG haplotype of rs1048661 and rs3825942) is associated with XFG in Japanese.

Our study supports association of the 82Gly allele and DR (P = 0.0003) with an odds ratio of 0.54 (95% confidence interval = 0.38 - 0.76) for the 82Ser (A) allele in a southern Indian population. Although the AA genotype appears to have an increased OR in the exploratory cohort (1.04, Table 2), this is due to the presence of a single AA individual in both the DR and DNR groups and probably represents a chance variation. Overall, the risk for DR decreases with the number of 82Ser (A) alleles, with both the additive model and dominant models supporting association. This supports the original findings of Kumaramanickavel et al. that 82Ser is a protective allele for diabetic retinopathy in the Indian population, and the similarities between the study populations in terms of ethnic origin and clinical criteria suggested that results from the subjects of both studies might be combined in a single analysis [21,33]. The individuals in the study by Kumaramanickavel et al. [21] were slightly older (63 years vs. 57 years average), had a slightly longer history of diabetes (18 years vs. 14 years average) and had somewhat higher HbA1C values (8.5 vs. 6.4 average). However, the similarities in the diagnostic and exclusion criteria and particularly the Indian ethnic composition of both groups allowed a combined analysis. The combined analysis was consistent with the results of subjects recruited from both hospitals, giving a similar odds ratios for the G allele (1.8 vs. 1.7), in the combined and ARAVIND patients respectively. The combined results, however, showed increased significance, with improvements in both the allelic (0.0003 vs. 0.003) whole SNP genotypic (0.0003 vs. 0.003) dominant (0.00011 vs 0.0013) and additive (0.00047 vs. 0.003) p-values in the combined and Aravind groups respectively. While we have not included a Bonferroni correction directly because of uncertainty about the a priori likelihood of each locus being true, a Bonferroni correction for multiple testing considering tests for 15 markers were performed might be a P < 0.0033, which is met in the combined ARAVIND and the final combined data sets.

In contrast to the results with rs2070600, neither the -374 T/A SNP nor the -429 T/C SNP in *RAGE *were found to be associated with DR, even though this association had been reported in the British population [34]. This might reflect population differences in the pathogenesis of DR in the Indian and other populations, but might also be a reflection of the relatively low minor allele frequencies in the Indian population, as the 95% confidence intervals for these SNPs, and especially -374 T/A, are very wide.

A putative transcription factor binding site of the *HTRA1 *gene in the 10q26 region was shown to confer significant risk for choroidal neovascularization in age related macular degeneration (AMD) and SNPs rs1061170, rs3753394 in *CFH *and rs10490924 in *ARMS2/LOC387715 *showed significant association [35-40]. Because neovascularization is common to both AMD and DR, we hypothesized that these genes may play a role in DR. To the best of our knowledge, this is the first report correlating the association of these SNPs with DR (however, our results indicate only marginal significance of the SNP rs11200638 in *HTRA1 *gene with DR).

We have attempted to avoid population substructure in our study. However, it remains possible that the positive and negative results could potentially be due to subtle population stratification and should be taken as suggestive rather than definitive.

## Conclusion

In conclusion, we are able to confirm association of the G82S polymorphism of *RAGE *with diabetic retinopathy in the population of southern India. We studied the association of SNPs rs11200638 in the *HTRA1 *gene, rs1061170, rs3753394 in *CFH *and rs10490924 in *ARMS2/LOC387715 *with DR for the first time. Our findings suggest that these three polymorphisms may not be associated with DR in the southern Indian population. Our data also strongly suggest that patients with the serine allele of the Gly82Ser (rs2070600) polymorphism in the *RAGE *gene show a decreased risk for diabetic retinopathy in the Indian population, although association of this allele with DR in a Chinese population suggests that this might represent linkage disequilibrium with a nearby causative rather than implying that glycation at this site plays a directly causative role in the development of diabetic retinopathy.

## Abbreviations

DR: Diabetic Retinopathy; DM: Diabetes Mellitus; T2DM: Type 2 Diabetes Mellitus; DNR: Diabetic patients without retinopathy; SNPs: Single Nucleotide Polymorphisms; RAGE: Receptor for Advanced Glycation End Products; PEDF: Pigment Epithelium Derived Factor; AKR1B1: Aldose Reductase; CFH: Complement Factor H; ARMS2: Age Related Maculopathy Susceptibility 2; EPO: Erythropoietin; HTRA1: High Temperature Requirement A1; ICAM: Intercellular Adhesion Molecule1; HFE: Hemochromatosis; OR: Odds Ratio.

## Competing interests

The authors declare that they have no competing interests.

## Authors' contributions

SB performed the sample collection, genotyping, statistical analysis, interpreted the data and drafted the manuscript. PS was involved in the study design and critically reading the manuscript. AR, KR and NP recruited and clinically characterized study subjects. GG was involved in genotyping of newly recruited samples. JFH was involved in the study design, data analysis, interpretation of the data, and preparation of the final version of the manuscript. All authors read and approved the final manuscript.

## Pre-publication history

The pre-publication history for this paper can be accessed here:

http://www.biomedcentral.com/1471-2350/11/158/prepub
